# Pre and Post-Operative Ph-Metry in Videolaparoscopic Surgery for Gastro Oesophageal Reflux Disease

**Published:** 2019-01-12

**Authors:** A Garzi, G Ardimento, U Ferrentino, S Brongo, R.M Di Crescenzo, E Calabrò, M. S Rubino, B Malamisura, E Clemente

**Affiliations:** 1 Division of Pediatric M.I.S. and Robotic Surgery University of Salerno, Italy; 2 Division of Pediatric Surgery University of Salerno, Italy; 3 Division of Plastic Surgery University of Salerno, Italy; 4 Division of Pathology University of Naples, Italy; 5 Pediatric Unit and Center for Celiac Disease-University Hospital of Salerno, Campus of Cava de’ Tirreni

**Keywords:** gastroesophageal reflux, videolaparoscopic surgery, pH-metry, outcome, follow-up

## Abstract

Gastro-oesophageal reflux is common in children, especially in the first year of life, and it may be regarded as physiological. Good functioning of the lower oesophageal sphincter depends largely on the anatomical relationships between oesophagus, stomach and diaphragm hiatus. Relative immaturity of these structures in newborn babies and young children is a risk factor in reflux disease, which may result in a wide variety of typical and/or atypical symptoms and, sometimes, serious complications such as oesophagitis and stenosis.

Reflux disease may be diagnosed and studied, basing on morphological and functional aspects and, since the advent of pH-metry, it is possible to personalise the therapeutic approach to children with reflux.

Surgical treatment of reflux disease in children has recently been improved due to a mini-invasive surgical approach. Absolute indications are recurrent pneumonia, intractable pain due to oesophagitis and retarded growth, often in association with neurological impairment.

In the last three years, 18 children with reflux disease underwent videolaparoscopic surgery in our department, 14 by the Nissen and 4 by the Toupet technique.

Post-operative pH-metry always showed a reduction in exposure of the distal oesophagus to acid (integral of H+) and an improvement in oesophageal clearance (short refluxes percentage) indicative of good functioning of the gastro-oesophageal junction.

PH-metry proved to be an invaluable technique for planning therapeutic strategy. In follow-up evaluations, it enabled us to monitor functioning of the gastro-oesophageal junction and to avoid other more difficult and invasive tests in patients with severe neurological impairment.

## I. INTRODUCTION

Gastro-oesophageal reflux disease is quite common in children. It has multifaceted aetiology and manifests in a variety of forms, depending on its amount, the chemical and physical properties of the refluxed material, oesophageal clearance, mucosal alterations, and reflex or mechanical effects of refluxed material on the airways.

Until recently, diagnosis was based solely on symptoms and upper digestive contrast X-ray. Lately, however, endoscopy and 24-hour pH-metry have been employed as new basic device. Analysis of 24-hour pH curve, mainly with respect to new generation parameters, has increased the accuracy of diagnosis, especially in cases in which X-ray and endoscopy are negative, and makes it possible to identify patients not responding to pharmacological therapy and requiring surgery.

The aim of this study was to evaluate 24-hour pH curve as a direct indicator of therapeutic efficacy in the follow-up of patients undergoing laparoscopic surgery.

## II. MATHERIALS AND METHODS

Eighteen children with reflux disease underwent videolaparoscopic surgery at the Section of Paediatric Surgery, Department of Paediatrics, of the University of Siena - Italy in the period 1998 – 2001. Indications for surgery were unsuccessful medical therapy and worsening of clinical symptoms and reflux. Six patients were excluded from the study as their post-operative follow up was not completed. The other twelve patients had a mean age of 6.08 years (age range 17 months to 16 years) at the time of the operation. Gender distribution did not show any significant statistical bias (7 males [58.3%], 5 females [41.7%]). Eleven patients had brain damage, with neurological and psychomotor impairment. These patients often had atypical symptoms, with recurrent airway infections, pneumonia and acute life-threatening events.

Pre-operatively, gastro-oesophageal transit, morphological alterations and gastro-oesophageal reflux were evaluated by upper digestive tract contrast X-ray. Gastroscopy was then performed to identify any malformations and assess the macroscopic and microscopic condition of serial oesophageal mucosa biopsy specimens.

Twenty-four-hour monitoring of gastro-oesophageal pH was used to evaluate reflux qualitatively and quantitatively. This method was originally standardised at our digestive pathophysiology unit. A computerised system (“pH-informer” - Deltron Electronics - Milan) was used with 1 second pH sampling, which is essential for bidimensional parameters evaluation (AUC, H+). Table 1 illustrates the results.

In nine cases (75.0%) videolaparoscopic Nissen fundoplication was performed; in four of these cases (44.4%) a temporary gastrostomy was necessary. In three cases (25.0%) a videolaparoscopic Toupet plasty was performed. Post-operative recovery was normal in all cases and no patient suffered from dysphagia.

All patients underwent follow-up, consisting of clinical examination, upper alimentary X-ray and pH-metry, within six months of the operation. Patients subsequently underwent clinical follow-up for a mean period of 17.7 months (range 7–30 months). Pre and post-operative pH data, expressed in medians (confidence intervals 25th–75th centile), were statistically compared by the Mann-Whitney test for repeated measurements (Table 2).

## III. RESULTS

Upper digestive tract X-ray showed abundant reflux in 7 cases (58.3%), and a cardio-tuberositary malpositioning in 3 cases (42.8%). Gastroscopy showed reflux in nine cases (75.0%), three (33,3%) with hiatal hernia and oesophagitis. PH-metry was positive in all cases with reflux, and traditional and new generation parameters had high values.

At follow-up, X-rays did not show any alteration in gastro-oesophageal transit and, from a functional point of view, distal oesophageal acid exposure and oesophageal clearance were normal (Table 4). Table 3 and table 4 show pre and post-operative values of gastro-oesophageal pH-metry. All parameters indicated a significant improvement in the function of the gastro-oesophageal junction after surgery.

## IV. DISCUSSION AND CONCLUSION

In patients with severe cerebral handicap, the first sign of reflux disease is often severe oesophagitis, which can be avoided if early pH-metric assessment is performed. This functional test provides an exact picture of the function of the gastro-oesophageal junction, through evaluation of distal true acid exposure. This is expressed by hydrogen ions concentration (mmol/l) and by oesophageal clearing capacity, expressed by the number of short refluxes (less than 0.5 seconds in duration) in the 24-hour period. In these patients reflux disease often manifests as an increase in convulsive crises, or respiratory symptoms unresponsive to specific therapy but responsive to anti-reflux therapy, or in severe cases, to surgical therapy.

PH-metry proved to be an invaluable technique for deciding therapeutic strategy. In follow-up evaluations, it enabled us to monitor functioning of the gastro-oesophageal junction and to avoid other more difficult and invasive tests in patients with severe neurological impairment.

## Figures and Tables

**Table 1:** Patients and pre-operative morphologic and functional studies.

ID	Age	Sex	X-ray	Gastroscopy	pH-metry
C.A.	7 ½ y	M	neg	Pos	Pos
B.M.	16 y	F	pos	Neg	Pos
S.G.	2 ½ y	M	pos	Neg	Pos
M.A.	3 ½ y	M	neg	Pos	Pos
G.J	4 ½ y	F	pos	Pos	Pos
F.A.	4 y	F	pos	Neg	pos
R.E.	17 y	F	neg	Pos	pos
A.F.	17 y	M	pos	Pos	pos
R.A.	12 y	M	pos	Pos	Pos
P.A	5 ½ y	M	neg	Pos	Pos
D.G.	8 ½ y	F	pos	Pos	Pos
M.R.	6 y	M	neg	Pos	Pos

**Table 2:** Mann-Whitney test: pH-metric parameters before and after surgery (bidimensional parameters in italics).

	T.T.R.	SCORE	RN	SR%	*A.U.C.*	*H+*
PRE	20.6 (12.7 – 41.8)	90.2 (48.9 – 139.2)	103.5 (82.0 – 146.0)	55.9 (39.1 – 60.6)	***281.4 (179.2 – 821.6)***	***1266.8 (523.2 – 6556.7)***
POST	0.35 (0 – 1.25)	2.2 (0.15 – 9.0)	10.0 (2 – 21)	93.2 (84.6 – 100)	***2.4 (1.3 – 3.3)***	***10.9 (8.8 – 27.1)***
P	<0,001	<0.001	<0.001	<0.001	***<0.001***	***<0.001***

**Table 3:** Preoperative pH-metry values (bidimensional parameters in italics)

ID	TTR (<4.2)	Score (<18)	RN (<50)	SR% (>60%)	*A.U.C. (<34)*	*H+ (<110)*
**M.A**	**10.8**	**58.5**	**85**	**38**	***122.0***	***316.759***
**S.G.**	**7.6**	**27.4**	**109**	**59**	***81.4***	***206.628***
**C.A.**	**43.8**	**185.3**	**155**	**38.7**	***897.5***	***8001.593***
**B.M.**	**87.6**	**232.4**	**29**	**36.5**	***1299.5***	***7804.010***
**G.J.**	**14.7**	**94.9**	**331**	**50.6**	***305.0***	***5309.447***
**F.A.**	**25.9**	**143.1**	**254**	**52.2**	***371.2***	***816.555***
**R.E.**	**5.8**	**21.4**	**56**	**56.1**	***45.6***	***176.892***
**A.F.**	**84**	**39.3**	**85**	**56.5**	***1941***	***10819.21***
**R.A.**	**14.7**	**79.9**	**98**	**55.4**	***236.5***	***729.7***
**P.A.**	**15.3**	**85.5**	**125**	**57.6**	***248.8***	***1717.172***
**D.G.**	**39.9**	**135.4**	**137**	**47.2**	***745.7***	***3987.87***
**M.R.**	**32.5**	**98.7**	**79**	**39.6**	***257.8***	***765.98***

**Table 4:** Post-operative pH-metry values (bidimensional parameters in italics)

ID	TTR (<4.2)	Score (<18)	RN (<50)	SR% (>60%)	*A.U.C (<34)*	*H+ (<110)*
**M.A.**	**0.0**	**0.0**	**1**	**100**	***0.2***	***26.875***
**S.G.**	**1.9**	**13.9**	**8**	**83.3**	***3.5***	***27.526***
**C.A.**	**0.6**	**4.6**	**43**	**86**	***10.2***	***58.519***
**B.M.**	**0.2**	**1.5**	**16**	**93.8**	***1.6***	***9.23***
**G.J.**	**0.0**	**0.0**	**3**	**100**	***3***	***10.237***
**F.A.**	**2.2**	**11.7**	**48**	**88**	***1.2***	***10.47***
**R.E.**	**0.5**	**2.2**	**8**	**66.7**	***3.1***	***3.894***
**A.F.**	**2.1**	**12.8**	**23**	**65.2**	***23.7***	***72.62***
**R.A.**	**0.0**	**0.3**	**1**	**100**	***1.3***	***11.456***
**P.A.**	**0.0**	**0.0**	**1**	**100**	***1.3***	***0.068***
**D.G.**	**0.0**	**2.2**	**12**	**96.4**	***2.6***	***22.67***
**M.R.**	**0.6**	**6.3**	**19**	**92.6**	***2.3***	***8.54***
